# West Nile Virus in *Culex* Mosquitoes in Central Macedonia, Greece, 2022

**DOI:** 10.3390/v15010224

**Published:** 2023-01-13

**Authors:** Katerina Tsioka, Sandra Gewehr, Styliani Pappa, Stella Kalaitzopoulou, Konstantina Stoikou, Spiros Mourelatos, Anna Papa

**Affiliations:** 1 Laboratory of Microbiology, National Reference Centre for Arboviruses, School of Medicine, Aristotle University of Thessaloniki, 54124 Thessaloniki, Greece; 2 Ecodevelopment S.A., 57010 Thessaloniki, Greece

**Keywords:** West Nile virus, *Culex pipiens*, Greece, lineage 2, whole-genome sequencing

## Abstract

In 2022, Greece was the second most seriously affected European country in terms of the West Nile virus (WNV), after Italy. Specifically, Central Macedonia was the region with the most reported human cases (81.5%). In the present study, 30,816 female *Culex pipiens* sensu lato mosquitoes were collected from May to September 2022 in the seven regional units of Central Macedonia; they were then grouped into 690 pools and tested for WNV, while next-generation sequencing was applied to the samples, which showed a cycle threshold of Ct < 30 in a real-time RT-PCR test. WNV was detected in 5.9% of pools, with significant differences in the detection rate among regional units and months. It is of interest that in the Thessaloniki regional unit, where most of the human cases were observed, the virus circulation started earlier, peaked earlier, and lasted longer than in the other regional units. All sequences clustered into the Central European subclade of WNV lineage 2, and the virus strain differed from the initial Greek strain of 2010 by 0.52% and 0.27% at the nucleotide and amino acid levels, respectively. Signature substitutions were present, such as S73P and T157A in the prM and E structural proteins, respectively. The screening of mosquitoes provides useful information for virus circulation in a region with a potential for early warning, while the availability of whole-genome sequences is essential for further studies, including virus evolution.

## 1. Introduction

West Nile virus (WNV) was initially isolated in 1937 from the serum of a febrile case in Uganda [1]. The virus belongs to the genus *Flavivirus* in the *Flaviviridae* family and possesses a single-stranded positive-sense RNA genome. WNV is maintained in nature, in an enzootic cycle between mosquitoes (mainly of the *Culex* genus) and birds [2]. Humans and equines are dead-end hosts due to the low-level viremia that they develop, which is not enough for further virus transmission [3]. Most cases (approximately 80%) of WNV infections are asymptomatic, and 20% present as mild cases of illness, while approximately 1% of the patients develop a neuroinvasive disease (mainly encephalitis, meningitis, or acute flaccid paralysis) [4,5,6].

Although up to nine of the WNV genetic lineages have been identified [7], the sequences in human cases cluster only into lineages 1 and 2 [8]. In Europe, the first human outbreak occurred in 1962 in southern France [9], while WNV lineage 2 was first detected in 2004 in Hungary and Russia (as independent virus introductions), with the sequences forming the Central European/Hungarian and the Eastern European/Russian clades of WNV lineage 2, respectively [10,11].

The first outbreak in Greece occurred in 2010; it started near a river delta in the Central Macedonia Region, in the northern part of the country [12]. A total of 197 neuroinvasive cases were reported, and 33 of them had fatal outcomes [13]. The strain responsible (Nea Santa-Greece-2010) was first detected in *Culex* spp. mosquitoes; phylogenetic analysis showed that the strain belongs to the Central European subclade of WNV lineage 2 [14,15]. Since then, WNV human cases have been reported every year, except in 2015 and 2016, while 2018 was a record year, with 243 cases showing neuroinvasive disease [16]. Furthermore, there are several reports on virus detection in mosquitoes, equines, and birds [17,18,19,20,21,22,23,24,25,26]. 

In 2022, Greece was the second most seriously affected European country, with 284 reported human cases of WNV, following Italy (586 cases) [27]. The aim of the present study was to screen for WNV *Culex pipiens* sensu lato mosquitoes, collected in 2022 in the Central Macedonia Region, and to perform phylogenetic analysis based on whole WNV-genome sequences. 

## 2. Materials and Methods

### 2.1. Mosquito Collection

A total of 30,816 female *C. pipiens* s.l. mosquitoes were collected from May to September 2022, in 139 sampling sites in the seven regional units of Central Macedonia, Greece (Chalkidiki, Imathia, Kilkis, Pella, Pieria, Serres, and Thessaloniki). Trapping was performed using CO_2_-baited light traps, with a constant CO_2_ outflow of 0.5 l/min, constructed by the Ecodevelopment mosquito control company (Thessaloniki, Greece). The selection of sites was based on environmental characteristics (e.g., terrain morphology—proximity to water bodies), and WNV detection in previous years. Additional traps were placed at 71 sites where new human cases were observed.

### 2.2. Mosquito Identification and Transportation 

The mosquito identification was performed using morphological identification keys [28,29]. The mosquitoes were transferred to the laboratory on dry ice and kept at −80 °C until processing. 

### 2.3. Laboratory Handling, RNA Extraction, and WNV Detection

The mosquitoes were grouped into 690 pools (max. 50 mosquitoes per pool), based on the trapping sites and collection dates (Table 1). The specimens were then washed with distilled water and homogenized in phosphate-buffered saline, using glass beads (diameter 150–212 μm) in a FastPrep FP120 cell disrupter (Bio-101, Thermo Savant; Q-Biogene, Carlsbad, CA, USA). The total RNA was extracted from 200 μl of supernatant of the homogenized pools, using the QIAamp cador Pathogen Mini Kit (Qiagen, Hilden, Germany) according to the manufacturer’s instructions. WNV was detected using a commercial real-time RT-PCR (RealStar WNV RT-PCR Kit, altona Diagnostics, Hamburg, Germany).

### 2.4. Next-Generation Sequencing and Phylogenetic Analysis

The samples that showed a cycle threshold (Ct) of less than 30 in the real-time RT-PCR test were chosen for further testing using a recently designed PCR-based next-generation sequencing (NGS) protocol [30]. The NGS libraries were prepared and quantified using the Ion Library TaqMan Quantitation kit; then, they were subjected to emulsion PCR on an Ion One Touch 2 system. After template enrichment, sequencing was performed on an Ion PGM sequencer, using a 316 Chip. Assembly and annotation were conducted in Geneious Prime, version 2021.2.1. The initial Greek sequence (HQ537483) was used as a reference. Whole-genome sequences were aligned with WNV lineage 2 sequences retrieved from the GenBank Database, and a maximum likelihood phylogenetic tree was constructed based on the best model, using MEGA version 11 software [31].

### 2.5. Maximum Likelihood Estimation, Minimum Infection Rate, and Statistical Analysis

The maximum likelihood estimation (MLE) value per 1000 mosquitoes for each regional unit was calculated using the PooledInfRate program, version 4.0 [32]. The minimum infection rate (MIR) was calculated as the ratio of positive pools to the total number of mosquitoes tested. A chi-square test was applied to determine whether the variation in the proportion of positive mosquito pools per regional unit and month was significant. The level of statistical significance was set to *p* < 0.001.

### 2.6. Data Visualization

The sites where mosquito trapping was performed were mapped using the geographic information system (GIS) application ArcGIS Pro, version 3.0 (Esri, Redlands CA, USA). 

## 3. Results

**Figure 1:**
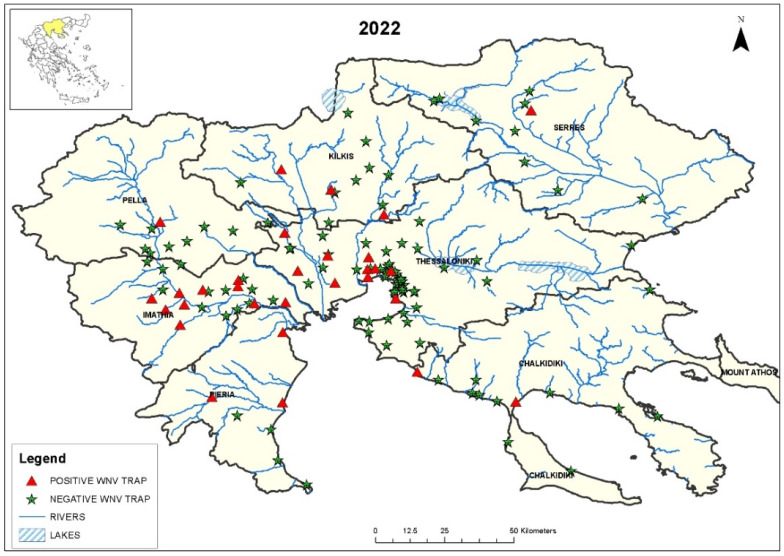
Map of Central Macedonia, with the sites where WNV-positive and -negative *C. pipiens* s.l. mosquitoes were trapped from May to September 2022. The location of the region in Greece can be seen in the inset in the upper left corner.

WNV RNA was detected in 41/690 (6.0%) *C. pipiens* s.l. mosquito pools (Table 1). The sites where the positive mosquito pools were detected in 2022 are shown in Figure 1. The rate of WNV-positive mosquito pools differed significantly per regional unit and month (*p* < 0.001). The virus was not detected in May; it started to be detectable in June (in the Thessaloniki regional unit), peaked in August, when positive mosquitoes were detected in all the regional units and continued to be detectable in September (in the Thessaloniki regional unit). 

The first WNV detection in the mosquitoes collected in the Central Macedonia Region was on 8 June 2022, in the regional unit of Thessaloniki (Table 2). Two weeks later, the first human case of the year was diagnosed in the same area [33]. In two additional regional units (Kilkis and Pieria), the virus detection in mosquitoes preceded the diagnosis of human cases (Table 2). The MLEs for the WNV infection rate per 1000 mosquitoes and the MIR per regional unit are shown in Table 3. In five regional units, the MLE and MIR values were >1. 

**Figure 2:**
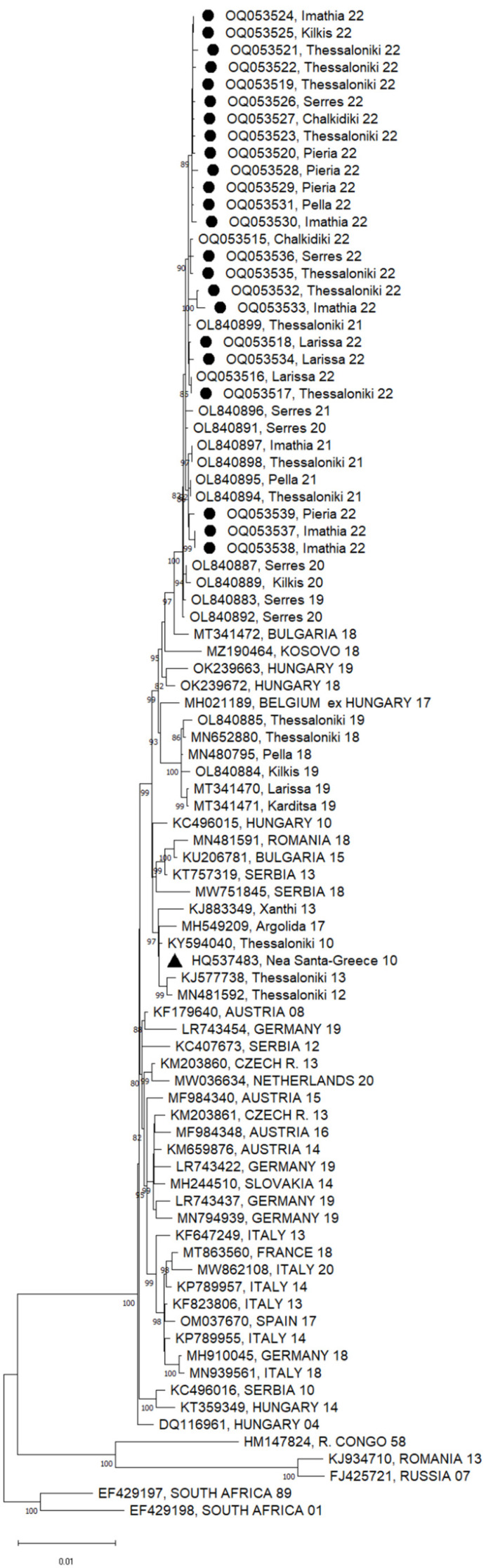
A phylogenetic tree based on the complete nucleotide sequences of the WNV lineage 2 polyprotein (10,302 nt) using the maximum likelihood method and Tamura–Nei model. A discrete gamma distribution was used to model the evolutionary rate differences among sites (5 categories (+ G, parameter = 0.2474)). The tree is drawn to scale, with branch lengths measured according to the number of substitutions per site. The percentage of trees in which the associated taxa are clustered together is shown next to the branches; only values >80% are shown. The tree is drawn to scale. The sequences of the present study are marked with a circle; the first Greek sequence (HQ537483) is marked with a triangle. The names of countries are shown in capital letters; all other sequences are from Greece.

Twenty-three of the 41 WNV-positive pools presented a Ct value of less than 30 in the real-time RT-PCR test and were subjected to NGS. A total of 3,270,641 reads were taken, and WNV sequences were recovered from all samples, with > 99.5% genome coverage. All sequences clustered within the Central European/Hungarian subclade of WNV lineage 2 (Figure 2). Sequences from all seven of the regional units are included. The nucleotide (nt) and amino acid (aa) differences among the sequences of 2022 were < 0.01%. It was seen that the sequences in 2022 form a distinct subclade, together with the Greek sequences from 2020 and 2021, along with one sequence from 2019 (OL840883). The nt and aa differences from the initial Greek WNV strain (HQ537483) were 0.52% and 0.27%, respectively. The closest published sequences from other European countries were those from Bulgaria (MT341472) and Hungary (OK239672), which were both detected in 2018 (differing from the Greek sequences of 2022 by 0.37% and 0.48 at the nt level, and 0.22% and 0.22% at the aa level, respectively). 

All the Greek sequences of 2020–2022, as well as one sequence from 2019 (OL840883), had an S73P substitution in the membrane glycoprotein precursor, prM, and a T157A substitution in the envelope glycoprotein. It is of interest that among the sequences included in the phylogenetic tree, the S73P substitution was present only in the sequences of the Eastern European subclade of WNV lineage 2 (KJ934710 and FJ425721). Furthermore, all sequences of 2021–2022, as well as one sequence from 2020 (OL840891), had a V106A substitution in the nonstructural protein, NS3. 

## 4. Discussion 

WNV infections constitute a serious threat to public health, especially to the elderly and immunocompromised persons. Since no WNV vaccine for humans is available, the only means of disease prevention is the limitation of mosquito bites. Prompt knowledge about the abundance of mosquitoes and their WNV infection rate in a particular region can direct decisions regarding the strategies needed for public and animal health prevention measures, including awareness among citizens, clinicians, and veterinarians, and mosquito control strategies. Therefore, mosquito surveillance and screening are useful tools by which to identify the areas with WNV circulation. 

Previous studies in Central Macedonia showed that the detection rate in mosquitoes ranged from 1.3% to 9.46% [17,23,24,34,35]. A plethora of biotic and abiotic factors affect the WNV circulation level, with temperature and precipitation having a major role since they influence the life cycle of the vectors [36]. The area where WNV emerged in Greece in 2010 was close to a major wetland area (the Axios Delta) which is on the migration route of millions of birds, while the plain of the wider area is one of the largest rice-producing areas in Europe [37], suggesting that there are many flooded fields of arable land in the area. Global phenomena, such as the North Atlantic oscillation (NAO) and El Niño southern oscillation events, might also influence the disease’s emergence and incidence; it was found that 2010 was a record year for the NAO, as the NAO index had previously had a continuously negative value (from October 2009 to January 2011) [38]. In the following years, the distribution and the incidence of WNV human cases in Greece varied, with a peak in 2018 (a record WNV year for Europe), when cases were spread all over the mainland of the country [16]. Another exceptional year was 2020, when Greece was the most affected country in Europe. Nearly half of the cases (56/144) occurred in the regional unit of Serres, where the WNV detection rate in mosquitoes was 14.3%; it was suggested that the upsurge in the virus circulation was probably related to anthropogenic factors that had an influence on the ecosystem of a major wetland in the area [39]. The situation was totally different in 2022; although again, the most affected region was Central Macedonia, the outbreak started in the regional unit of Thessaloniki, which has the highest human population in Central Macedonia (1,091,424 in the census of 2022). There, the WNV detection in mosquitoes started earlier, peaked earlier, and lasted longer than in other units of Central Macedonia (Table 1). The longest virus circulation in Thessaloniki resulted in 115 human cases (out of 233 in the whole region) [33]. Specifically, the first cases were reported in the city of Thessaloniki (the largest city in the region, with a population of 814,000 in the census of 2022), instead of in the rural areas, and cases were continuously reported in the city until October. A factor that might have played a role could be the repeated heavy rainfalls during June, which led to an increased number of *C. pipiens* s.l. breeding sites in the urban system and, at the same time, made the mosquito control efforts less effective, due to the flushing out of the insecticides applied in the rainwater catchment basins. 

Mosquitoes, horses, birds, and other animals can be used as sentinel species for WNV surveillance. Previous studies in Greece showed that in some cases, the virus was detected earlier in mosquitoes (by approx. two weeks) than in human cases [23,24]. This was also seen in 2022, as the first virus detection in mosquitoes in the country preceded the diagnosis of the first human case by two weeks, serving as an early warning system. The MLE and MIR values were > 1 in five of the regional units, and the early warning worked in three of them (Thessaloniki, Kilkis, and Pieria) (Table 2 and Table 3). 

Since 2010, when the virus emerged in Greece, all sequences cluster into the Central European subclade of WNV lineage 2, except for one case in 2018 that was caused by a strain belonging to the Eastern European subclade [40]. The sequences of the present study constituted a distinct subclade, being evolutionary variants of a strain introduced into the country in 2019 (OL840883). Specific substitutions characterized the sequences of 2020–2022; it is not known whether they play any role in the biological properties of the strains. The availability of more sequences, covering a broad spatial and temporal distribution of WNV strains in Europe, will lead to accurate phylogenetic and phylogeography studies. 

## 5. Conclusions

An intense WNV circulation occurred in 2022 in Greece, especially in the Central Macedonia Region, where 5.9% of the tested *C. pipiens* s.l. pools were positive. The screening of mosquitoes provides information about virus circulation in an area, which is useful for both veterinary health and human public health, while the availability of whole-genome sequences is the basis for further studies, including those on virus evolution. 

## Figures and Tables

**Table 1:** Detection of West Nile virus in *Culex pipiens* s.l. mosquitoes, shown per month and regional unit of Central Macedonia, Greece, 2022.

Regional Unit	May		June		July		August		September		Total	
	No. Pools	No. Pos. Pools (%)	No. Pools	No. Pos. Pools (%)	No. Pools	No. Pos. Pools (%)	No. Pools	No. Pos. Pools (%)	No. Pools	No. Pos. Pools (%)	No. Pools	No. Pos. Pools (%)
**Chalkidiki**	10	0 (0)	7	0 (0)	6	0 (0)	13	2 (15.4)	9	0 (0)	45	2 (4.4)
**Imathia**	13	0 (0)	15	0 (0)	24	4 (16.7)	28	6 (21.4)	12	0 (0)	92	10 (10.9)
**Kilkis**	9	0 (0)	6	0 (0)	7	1 (14.3)	8	3 (37.5)	17	0 (0)	47	4 (8.5)
**Pella**	4	0 (0)	6	0 (0)	7	0 (0)	14	1 (7.1)	7	0 (0)	38	1 (2.6)
**Pieria**	7	0 (0)	8	0 (0)	11	1 (9.1)	14	3 (21.4)	9	0 (0)	49	4 (8.2)
**Serres**	13	0 (0)	11	0 (0)	16	0 (0)	15	1 (6.7)	17	0 (0)	72	1 (1.4)
**Thessaloniki**	69	0 (0)	64	3 (4.7)	72	10 (13.9)	78	2 (2.6)	64	4 (6.2)	347	19 (5.5)
**Total**	**125**	**0 (0)**	**117**	**3 (2.6)**	**143**	**16 (11.2)**	**170**	**18 (10.6)**	**135**	**4 (3.0)**	**690**	**41 (5.9)**

**Table 2:** Dates of WNV detection in mosquitoes and humans, per regional unit of the Central Macedonia region, Greece, 2022.

Regional Unit	First Detection in Mosquitoes	Diagnosis of First Clinical Cases
Chalkidiki	8 August	28 July
Imathia	26 July	12 July
Kilkis	6 July	18 July
Pieria	18 July	2 August
Serres	18 August	3 August
Pella	24 August	18 July
Thessaloniki	8 June	23 June

**Table 3:** MLE and MIR values per regional unit of Central Macedonia, Greece, 2022.

Regional Unit	Mosquitoes	Pools	Positive Pools	MLE (95% CI)	MIR (95% CI)
Imathia	5271	92	10	2.04 (1.03–3.61)	1.90 (0.72–3.07)
Thessaloniki	13507	347	19	1.48 (0.92–2.26)	1.41 (0.77–2.04)
Kilkis	1678	47	4	2.5 (0.81–5.89)	2.38 (0.05–4.72)
Pieria	2638	38	1	1.5 (0.48–3.55)	1.44 (0.03–2.85)
Pella	2779	49	4	0.39 (0.02–1.86)	0.38 (0–1.12)
Serres	3472	72	1	0.29 (0.02–1.4)	0.29 (0–0.85)
Chalkidiki	1553	45	2	1.34 (0.24–4.35)	1.29 (0–3.07)
**Total**	30,898	690	41	**1.39 (1.01–1.86)**	**1.33 (0.92–1.73)**

## Data Availability

Nucleotide sequences identified in the study were submitted to the GenBank DataBase under the accession numbers OQ053517–OQ053539.
